# Multiple myeloma in Nigeria: a multi-centre epidemiological and biomedical study

**DOI:** 10.11604/pamj.2015.22.292.7774

**Published:** 2015-11-24

**Authors:** Odunukwe Nkiruka Nnonyelum, Madu Jude Anazoeze, Nnodu Obigeli Eunice, Okocha Onyichide Emmanuel, Akingbola Titilola Stella, Asuquo Inyama Marcus, Balogun Modupe Taiwo, Kalejaiye Olufunto Olufela, Aneke John Chinawaeze, Joseph Aondowase Orkuma, Gwarzo Gwarzo Dalhat, Ujah Innocent Otobo

**Affiliations:** 1 Clinical Sciences Division, Nigerian Institute of Medical Research Lagos Nigeria; 2 Department of Haematology and Blood Transfusion, University of Nigeria Teaching Hospital Enugu, Nigeria; 3 Department of Haematology College of Health Sciences University of Abuja teaching Hospital, Nigeria; 4 Department of Haematology and Blood Transfusion, Nnamdi Azikiwe University Teaching Hospital Nnewi, Nigeria; 5 Department of Haematology, Faculty of Basic Medical Sciences, University of Ibadan; 6 Department of Haematology and Blood Transfusion University of Calabar Teaching Hospital Calabar, Nigeria; 7 Department of Haematology and Blood Transfusion, Lagos State University Teaching Hospital Ikeja, Nigeria; 8 Department of Haematology, College of Health Sciences, Benue State University, Makurdi-Benue State; 9 Department of Haematology and Blood Transfusion University of Sokoto Teaching Hospital Sokoto, Nigeria

**Keywords:** Multiple myeloma, Nigeria, presentation, treatment modalities

## Abstract

**Introduction:**

Myelomatosis is a malignant proliferation of plasma cells in the bone marrow, with relatively high prevalence in African populations. Variation in genetic mutations has been observed in individual patients and may be responsible for differences in disease pattern and treatment outcomes. This study described the presentations and treatment outcomes of multiple myeloma in nigerian.

**Methods:**

The data was obtained retrospectively from the case notes of 135 patients diagnosed with multiple myeloma from eight tertiary health institutions across the six geopolitical zones of Nigeria from 2005 to 2014. Data analysis was carried out using SPSS 17.0.

**Results:**

The predominant presentations were bone pain in 97 (74%), nephropathy in 47 (35.9%) and pathological fractures in 58 (44.3%). Sixty-seven percent (67%) of the patients were less than 60 years, and 35% had Bence Jones proteinuria. The overall survival beyond 6 months was 91.3%, mean duration of survival rate was 7.4 months. Majority (66.2%) were on Melphalan alone or on melphalan-containing combinations. A higher packed cell volume (PCV) and total serum protein levels at presentation were associated with increased survival, p=0.033 and 0.036, respectively.

**Conclusion:**

This study portrayed the importance of detail investigation on the causes of bone pain and anaemia in person's aged 40 years and above. There is a high prevalence of nephropathy in this cohort of patients which needs to be further investigated. Majority of the patients, though < 65 years of age were placed on melphalan-containing combinations, which foreclosed chances of future autologous bone marrow transplantation.

## Introduction

Multiple myeloma (MM) refers to the monoclonal proliferation of bone marrow plasma cells with attendant bone disease and clinical features associated with marrow invasion. There is marked variability in the clinical features seen in this patient population, from the apparently healthy patients to the debilitated ones with anaemia and pathological fractures, with or without nephropathy [1].

The Haematological Malignancy Research Network (HMRN) [2] region for 2004-2009 described lower rates of myeloma incidence in the more deprived areas for both sexes combined while the England-wide data for 2000-2004 earlier documented a similar rate only in females [3]. These observations are compatible with the theory that socio-economic factors impact on the likelihood of recognizing the non-specific symptoms common in myeloma which inform the attitude to seeking medical care [4, 5].

Previous data from the Statistics, Epidemiology and End Result (SEER) program showed a significantly better survival in blacks with MM, compared with whites [6, 7]. Though monoclonal, several mutations have been associated with the malignant transformation as well as prognosis of this disease, a recent study has shown variations in the prevalence of t(11;14), t(4;14), monosomy 13/del13q and monosomy 17/del17p, with blacks having lower figures [8].Clinical features as well as outcomes of treatment are known to vary according to the underlying genetic mutations and thus confer unique disease features. Chemotherapeutic options in diagnosed cases of MM involve patient stratification based on age, presence of co-morbidities and peculiar mutations detected in each patient [9]. In the African setting, challenges in the aspect of patients? investigative assessment as well as affordability and access to first line medications and transplant facilities are quite limited. This invariably affects treatment outcomes and survival. Previous single centre study conducted by Madu *et al* in southeastern Nigeria placed duration of follow up at 24 weeks [10].

This is a multi-centre retrospective study aimed at providing a broader assessment of clinical and biomedical features of patients with MM in Nigeria. Therapeutic choices and treatment outcomes among this patient population were also analysed for evidence-informed management.

## Methods

The data was obtained retrospectively from the case notes of 135 patients diagnosed with multiple myeloma from eight tertiary health institutions across the six geopolitical zones of Nigeria from 2005 to 2014. The information obtained included; clinical and laboratory features at presentation, chemotherapeutic regimen used, mortality and duration from diagnosis to death or last appointment. Each centre obtained ethical approval from their institution in addition to the approval obtained from the Nigerian Institute of Medical research Institute Review Board (IRB).

Data analysis was carried out using SPSS 17.0 (IL, Chicago), and Spearmann as well as Kendaulltau_b to assess relationship between nominal and ordinal data. Kaplan -Meier survival was estimated and the curve was plotted using the same software. Values were assumed to be significant if p value was ≤0.05.

## Results

Of the 135 patients studied, 70(58.9%) were males and 65 (48.1%) were females. The median age of the patients was 60 years and a mean 58.8 ± 11.2 years. Bone pain was the presenting feature in 97 (74%) of the patients, while 58 (44.3%) of the patients had pathological fracture at presentation and 47 (35.9%) had nephropathy. Table 1 shows the distribution of the patients among the participating centres. Hypertension was observed as a predominant co-morbidity in 60% (15/25) of patients.

**Table 1:** Age group distribution

<40	5
40-50	19
51-60	29
61-70	34
>70	13
total	100

Majority (77%) of the patients were moderate to severe anaemia at presentation with a median haemoglobin concentration of 8.3g/dL, and a mean value of 8.4 ± 2.1g/dL. The median values of the total protein, albumin and globulin were found to be 7.6, 3.4 and 4.7g/L, respectively. Bone marrow plasmacytosis was observed in all patients with a median percentage of 28.5%, and serum calcium had a median value of 2.7mmol/L, and a mean value of 5.0 ± 5.5 mmol/L. Of the 68 patients assessed for Bence Jones proteinuria, 24 (35.3%) were positive while 44 (64.7%) were negative. Serum protein electrophoresis showed a monoclonal gamma band in 38 (45%) of the patients, but absent in 66 (55%) of cases.

**Figure 1:**
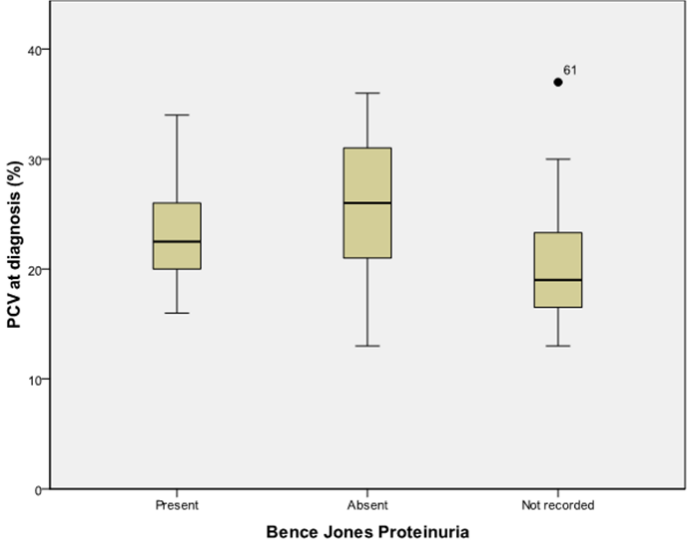
Haemoglobin concentration and presence of Bence Jones proteinuria

**Figure 2:**
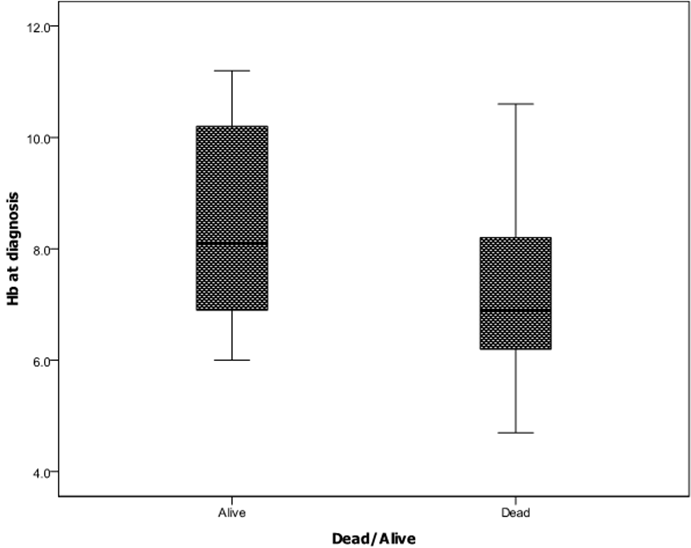
Relationship between haemoglobin concentration at diagnosis and patient mortality

There was a significant relationship between the occurrence of pathological fractures and a lower serum calcium level with a correlation coefficient of 0.267 (p=0.004).There was also a significant relationship between increasing age and the presence of Bence Jones proteinuria (BJP) and nephropathy (correlation coefficient of 0.229 (p=0.039) and 0.144 (p=0.046)) respectively.The serum globulin level is also associated with the presence of BJP and percentage of bone marrow plasmacytosis; 0.252 (p=0.012) and 0.368 (p=0.001), respectively. Patients who had a higher PCV at presentation seemed to have a higher probability of not excreting BJP in urine as shown in Figure 1. A higher total serum protein value was also associated with increased survival coefficient of 0.190 (p=0.036). Patients with a higher PCV at presentation were also noted to survive longer, coefficient of 0.202 (p=0.033) and as shown in Figure 2.

**Figure 3:**
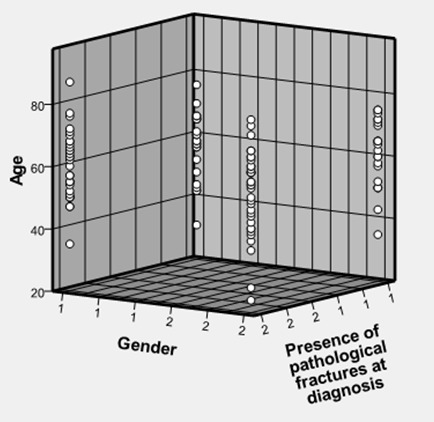
Occurrence of pathological fractures in the various gender and ages of patients

**Figure 4:**
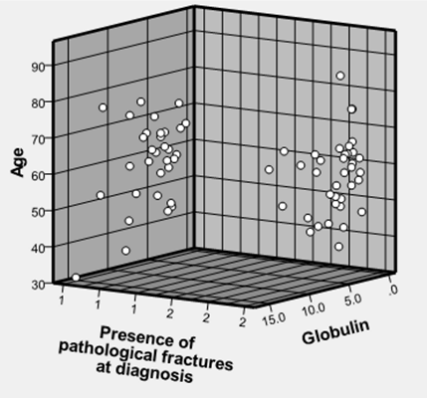
Age and serum globulin distribution in patients with or without pathological fractures

**Figure 5:**
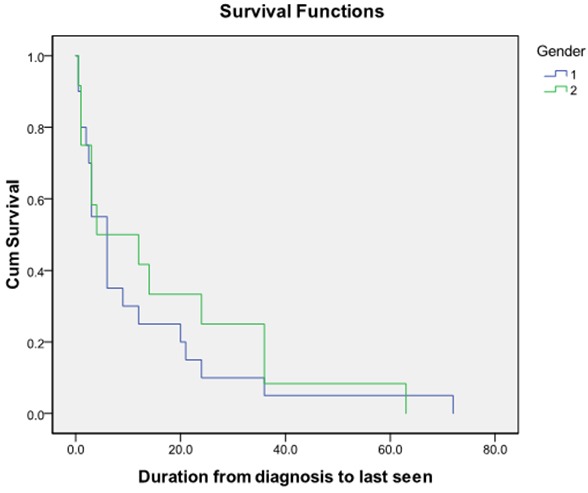
Kaplan Meier survival curve in males and females with myeloma

Pathological fractures as depicted in Figure 3 do not seem to occur more in the older patients, nor do they seem to be more prevalent in any particular gender.However the prevalence of pathological fractures seems to be fairly well distributed across the various ages irrespective of their serum globulin levels (Figure 4). Males seem to survive longer than females (Figure 5).

A great number (48.6%) of the patients received melphalan-based combinations (melphalan/prednisolone (MP) 30.6% and melphalan/prednisolone/ thalidomide (MPT) 18.4%). Only 5 (13.9%) patients aged less than 65 years received the first line drug Bortezomib in combination with other drugs.

## Discussion

The demographic variables in this study were found to be similar to those obtained in other studies on multiple myeloma patients in Nigeria by Madu *et al* [10] and Fasola *et al* [1]; majority being females with median age of 60 years. Bone pain seemed to predominate as a presenting feature and in most cases associated with anaemia. Hence the occurrence of these features in a patient aged 40 years and above should raise a high index of suspicion for multiple myeloma. Our results also indicated that though Bence Jones proteinuria (BJP) was seen in some patients, a significant number of patients were negative for Bence Jones proteinuria. This may be a possible outcome of the variations in the genetic mutations responsible for initiating this malignancy, which has been observed across the different races as recently reported by Greenberg *et al* [11]. The reliance on the BJP and serum protein electrophoresis as a diagnostic protocol, may lead to several missed cases, as the results of this study have shown their low specificity.

Pathological fracture is a major cause of increased morbidity in patients with multiple myeloma and could predispose to paraplegia with adverse effects on survival [12]. This complication was present in 58 (44.3%) of cases in this study and did not correlate with age of patients (Figure 3). Even though fractures increase with age in the general population [13], this may not be the case in patients with multiple myeloma as fractures may be an indication of bone disease and not necessarily bone ageing.

Hypercalcaemia is a frequent metabolic complication in patients with multiple myeloma; occurring in up to 1/3 of cases[14]. It is closely linked with the development of myeloma bone disease, particularly pathological fractures, and stems from loss of bone density due to osteolysis, with efflux of calcium into the extracellular fluid. Bone resorption in patients with multiple myeloma is thought to be mediated by a number of cytokines, including receptor activator of nuclear factor kappa -B ligand (RANKL), macrophage inflammatory protein (MIP) 1α and tumour necrosis factors (TNFs) which are either secreted by the myeloma cells or by other accessory cells involved in cell-cell interactions within the marrow micro-environment [15]. In spite of the reported high frequency of hypercalcaemia and its association with myeloma bone disease, this study observed that lower serum calcium levels were positively associated with occurrence of pathological fractures. While this may appear contradictory, other reports have noted that hypercalcaemia may not be consistently associated with myeloma bone disease and may even be a late (terminal) feature of multiple meloma [14, 16]. These reports showed that hypercalcaemia was not present in all myeloma patients that had significant bone disease and surmised that the relationship between hypercalcaemia and myeloma bone disease may not be limited to osteoclastic activation (with increased bone resorption) but could involve other influences such as those related to myeloma cell mass, glomerular filtration status and disease duration. It may thus appear that these (other) influences predominate in our patients and could account for the positive relationship observed between low serum calcium and the occurrence of pathological fractures in this study.

A little less than half of the patients had already developed nephropathy at the point of diagnosis. Similar prevalence of this complication had been reported by previous authors. The study at Ibadan by Fasola *et al* reported that about one-third of the patients had renal failure while a further two-thirds had renal insufficiency [1]. It should be further investigated if this was a consequence of late presentation or a peculiar feature of MM in Nigeria. Other factors such as presence of co-morbidities (especially hypertension) should be ruled out as contributing to the increase in prevalence of renal dysfunction. Also other known causes of nephropathy in MM such as amyloidosis, hypercalcaemia, anaemia and recurrent urinary tract infections should be vigorously investigated in all suspected cases. According to the study by Fasola *et al* [1], hypercalcaemia was a common feature of patients who had developed renal failure compared with those that had normal renal function.

The presence of Bence Jones protein in urine at presentation seems to have some interesting associations with; age, serum globin level and percentage of marrow plasmacytosis. These findings can be explained to a large extent from the fact that the protein is produced by the malignant plasma cells and occupies the globin fraction of the plasma proteins. Sinohara *et al* reported that approximately 50% of BJP are catabolized in the kidneys [17]. Therefore, its occurrence with increasing age seems to infer that the renal reabsorption capacity of the protein diminishes with age and therefore Bence Jones proteinuria should be expected in the older patients with MM.

A higher serum protein level as well as PCV was associated with improved survival. These parameters are usually used in assessing degree of well-being in chronically ill patients. These patients are therefore more likely to tolerate chemotherapy and withstand infections better. Whether this can be translated to mean that optimizing the PCV and serum proteins always in MM patients would markedly improve survival, has to be further evaluated. This may also imply that dietary considerations may play a major role in the management of patients in this setting, as had been suggested in previous publications [6, 18]. It remains to be evaluated whether increasing the protein (albumin) content of diet or optimizing the haemoglobin concentration as part of the management protocol, in MM will improve survival.

Majority of the patients across the various participating centres received melphalan either alone or in combination with other cytotoxic drugs, irrespective of age or eligibility for bone marrow transplants (BMT). This falls short of the current best practice with regard to management of MM. Financial constraints and lack of availability of first choice drugs for use in younger patients has limited the treatment options available to haem-oncologists in Nigeria. The increased survival and relapse rate obtained with the newer drugs may need to be replicated in this patient population as satisfactory results have been obtained with alternative combinations. This further underlines the need to characterize the predominant cytogenetic mutations in this cohort in order to suggest a well suited and better tailored regimen.

## Conclusion

The importance of having a high index of suspicion of MM in patients aged 40 years and above presenting with bone pain and anaemia cannot be over-emphasized. The predictive factors for the causation of nephropathy in MM need to be further evaluated to help prevent this predominant complication which negatively affects survival. A higher haemoglobin concentration and serum protein positively affect survival and this should be exploited in aspects of management. Most of the patients were placed on melphalan-containing combinations, irrespective of their age. Newer, first line drugs should not only be made available, but also tried in this peculiar group of patients.
